# The spectrum of factor XI deficiency in Southeast China: four recurrent variants can explain most of the deficiencies

**DOI:** 10.1186/s13023-024-03235-5

**Published:** 2024-06-04

**Authors:** Ke Zhang, Langyi Qin, Fei Xu, Longying Ye, Mengzhen Wen, Jingye Pan, Lihong Yang, Mingshan Wang, Haixiao Xie

**Affiliations:** 1 Department of Clinical Laboratory, Key Laboratory of Clinical Laboratory Diagnosis and Translational Research of Zhejiang Province, The First Affiliated Hospital of Wenzhou Medical University, Wenzhou, 325000 China; 2 Department of Intensive Care Unit, The First Affiliated Hospital of Wenzhou Medical University, Wenzhou, 325000 China

**Keywords:** Inherited bleeding disorders, Factor XI deficiency, Variant, Gene frequency

## Abstract

**Background:**

Factor XI (FXI) deficiency is an autosomal hemorrhagic disorder characterized by reduced plasma FXI levels. Multiple ancestral variants in the *F11* gene have been identified in Ashkenazi Jews and other selected European populations. However, there are few reports of predominant variants in Chinese and/or East Asian populations. The aim of this study is to characterize the genotypes and phenotypes of FXI deficiency and identify the predominant variants.

**Results:**

Of the 41 FXI-deficient patients, 39 exhibited severe FXI defects, considerably more than those with partial defects. The APTT levels showed a negative correlation with FXI activity levels (coefficient=-0.584, *P* < .001). Only nine patients experienced mild bleeding, including one partially defective patient and eight severely defective patients. The majority of patients were referred for preoperative screenings (*n* = 22) and checkups (*n* = 14). Genetic analysis revealed that 90% of the patients had genetic defects, with 2, 16, and 19 cases of heterozygous, homozygous, and compound heterozygous patients, respectively. Seventeen variants were detected in the *F11* gene (6 novel), including eleven missense variants, four nonsense variants, and two small deletions scattered throughout the *F11*. Of the 11 missense variants, six have not yet been studied for in vitro expression. Protein modeling analyses indicated that all of these variants disrupted local structural stability by altering side-chain orientation and hydrogen bonds. Nine variants, consisting of three missense and six null variants, were detected with a frequency of two or more. The highest allele frequency was observed in p.Q281* (21.25%), p.W246* (17.50%), p.Y369* (12.50%), and p.L442Cfs*8 (12.50%). The former two were variants specific to East Asia, while the remaining two were southeast China-specific variants.

**Conclusion:**

Our population-based cohort demonstrated that no correlation between the level of FXI activity and the bleeding severity in FXI deficiency. Additionally, the prevalence of FXI deficiency may have been underestimated. The nonsense p.Q281* was the most common variant in southeast China, suggesting a possible founder effect.

## Introduction

Coagulation factor XI (FXI) is a serine protease, approximately 160 kDa in size, primarily synthesized by the liver. When activated by factor XII (FXII), thrombin, or self-cleavage, FXI can be converted to activated FXI (FXIa). This process activates factor IX and exerts a hemostatic effect through the intrinsic coagulation pathway [1]. After intracellular processing, each FXI monomer comprises 607 amino acids, including four apple domains (Ap1 to Ap4 from the N-terminus) comprising 90 or 91 amino acids and a C-terminal serine-containing catalytic domain (SP) for serine protease activity [2, 3]. FXI circulates in human plasma as a homodimer composed of a unique two-subunit structure at a concentration of approximately 30 nM (15–45 nM). This dimeric structure is crucial for the efficient secretion and activation of the FXI protein [4].

The *F11* gene (GenBank accession number, NG_008051.1) encoding FXI is located at the long arm of chromosome 4 (4q35) and comprises 15 exons along with 14 introns (A-N). Hereditary FXI deficiency (OMIM#264,900) is an autosomal recessive or dominant bleeding disorder caused by a defect in the *F11* gene. The variants in the *F11* gene are often categorized as cross-reactive material-negative (CRM-) or cross-reactive material-positive (CRM+) based on the presence or absence of dysfunctional proteins in plasma, characterized by synchronously reduced FXI activity (FXI: C) and antigen (FXI: Ag) and low FXI: C and normal FXI: Ag, respectively [5].

FXI deficiency may cause mild to moderate bleeding, which manifests primarily from trauma or surgical hemostatic challenges. FXI is the sole contact factor involved in physiologic coagulation, as there is compelling evidence that defects in FXII, prekallikrein (PK), and high molecular weight kallikreinogen (HMWK) do not produce a bleeding phenotype [6]. This may be attributed to a positive feedback effect stemming from the fact that FXI is also activated by thrombin. However, clinical heterogeneity exists in the presentation of FXI-deficient patients, and the correlation between genotype and phenotype tends to be weak [7]. Most FXI-deficient patients do not exhibit significant bleeding symptoms [8]. Hence, patients are usually diagnosed incidentally, for instance, during preoperative or physical examination screening. Therefore, many studies have shown that FXI inhibitors can serve as targets for anticoagulant therapy, ultimately preventing venous thrombosis and potentially treating atherosclerosis and hypertension [9, 10]. A few patients also experience unprovoked bleeding, primarily in mucous membranes, including nosebleeds, menorrhagia, and gingival bleeding, and these bleeding sites have high fibrinolytic activity. It has been shown that FXI can exert antifibrinolytic effects by activating thrombin-activated fibrinolytic inhibitor (TAFI) [11].

The objective of this study was to investigate the correlation between phenotype and genotype and summarize the southeast China-specific variant spectrum of the *F11* gene in 41 patients with FXI deficiency.

## Materials and methods

### Subjects, blood collection, and coagulation tests

This retrospective cohort study was approved by the Ethics Committee of the First Affiliated Hospital of Wenzhou Medical University, Wenzhou, China. A total of 41 unrelated index patients who received treatment at our hospital from 2016 to 2023 were included in the study. Patient demographic details, including age, sex, family history, bleeding history, and consanguinity, were documented. Patients with acquired FXI deficiency and a history of liver and kidney disease were excluded. The bleeding scores for all subjects were calculated using the International Society on Thrombosis and Haemostasis Bleeding Assessment Tool (ISTH-BAT, https://practical-haemostasis.com). Peripheral venous blood was obtained from all patients using siliconized glass tubes containing sodium citrate anticoagulant. The upper layer of poor-platelet plasma was utilized for routine coagulation screening after centrifugation at 3000 xg for 10 min. The prothrombin time (PT), activated partial thromboplastin time (APTT), fibrinogen, and FXI: C were detected using a commercial kit by the clotting method on the STAGO STA-R-Max automatic blood analyzer (Diagnostica Stago, Seine-et-Marne, France). Informed consent was obtained from all participants.

### Genomic DNA extraction and variant screening

According to the manufacturer’s instructions, leukocytes from participants were utilized for extracting genomic DNA via QIAamp DNA Blood Kits (GIAGEN, Hilden, Germany). All 15 exons of the *F11* gene, along with a minimum of 100 base pairs of intron flanking sequence, were amplified by the polymerase chain reaction (PCR) on the Thermocycler 2720 (Applied Biosystems, California, USA). The PCR amplification procedure entailed pre-denaturation at 95 °C for 5 min, followed by denaturation at 95 °C for 30 s, annealing at 55 °C for 30 s, and extension at 72 °C for 30 s, totaling 30 cycles. The final extension at 72 °C for 10 min concluded the procedure. All PCR primers were designed using Primer-BLAST (https://www.ncbi.nlm.nih.gov/tools/primer-blast/) as previously described [12]. The PCR amplification products were purified and directly sequenced by Saiheng Bio-Technology Corporation (Shanghai, China). Potential mutant sites were identified using Chromas software (Chromas *v*2.23), and these sites were confirmed through reverse sequencing and/or clone sequencing. When unreported variants were detected, a total of 50 healthy individuals (100 alleles) were randomly selected as controls to exclude the polymorphism. According to the Human Genome Variation Society (HGVS) variant nomenclature guide [13], the amino acid numbering included the presence of the signal peptide (starting at Met1), with NM_000128.4 as the RefSeq. Subjects with homozygous or compound heterozygous variants typically exhibit less than 20% FXI: C and are classified as severe deficiency. Those with heterozygous variants are classified as partial deficiency [14, 15].

### *In silico* analysis

The degree of conservation in *Homo sapiens* and five other mammalian species, including *Canis lupus familiaris, Bos taurus, Pan troglodytes, Macaca mulatta, and Mus musculus* (HomoloGene, http://www.ncbi.nlm.nih.gov/homologene), was estimated using ClustalX-2.1-win software. The pathogenicity of the nonsynonymous missense variants was predicted through the VarCards (http://www.genemed.tech/varcards/). The PyMOL software was utilized to evaluate the impact of in vitro uncharacterized missense variant s on the structure of the FXI protein and to visualize the protein. The FXI protein crystal structure data were obtained from the Protein Data Bank (PDB, https://www.rcsb.org/, PDB ID: 5EOK).

### Statistical analysis

All the statistical analyses were performed using SPSS software version 16.0 for Windows (IBM, Armonk, USA). Quantitative information is expressed as the median. In addition, a nonparametric Spearman’s rank correlation test was conducted on both data sets to demonstrate the correlation between APTT and FXI: C levels. Correlation coefficients were recorded, and the p-value < 0.05 was considered to indicate statistical significance.

## Results

### Patients

Forty-one consecutive unrelated patients (14 males and 27 females) were examined (Table 1). The ages ranged from 9 to 86 years, with a median age of 48.5 years. The laboratory tests revealed that both the APTT and FXI: C were outside the reference range. The median APTT was 82.6 s, while the median FXI: C was 3%. Of the patients studied, 39 had severe FXI defects, which was significantly greater compared to those with partial defects. The Spearman’s rank correlation test revealed a negative correlation between APTT levels and FXI: C levels (coefficient=-0.584, *P* < .001). The majority of patients were referred for preoperative screening (*n* = 22, 53.66%) and check-ups (*n* = 14, 34.15%). In addition, five women were diagnosed with severe FXI defects, including three during antenatal examination and two due to hemostatic difficulties. Of the total cases, 32 patients did not exhibit any bleeding symptoms. Among the cases where abnormal bleeding occurred (*n* = 9), four cases resulted from bleeding after surgery or trauma. Additionally, three cases involved menorrhagia while nosebleed, gingival bleeding, and urinary tract bleeding each presented once. The median ISTH-BAT bleeding score for these nine patients was 2, and all bleeding was mild. One of only two patients with partial FXI defects (Patient 2, P2) exhibited postoperative bleeding. Furthermore, while P3 did not experience any abnormal bleeding, his brother who was partial defect (FXI: C level of 48%) did experience postoperative bleeding. Four patients (P3, P23, P25, P30) received preoperative supplementation with fresh frozen plasma (FFP), and none of them experienced abnormal postoperative bleeding.

**Table 1:** The phenotype and genotype of the 41 FXI-deficient patients

Case No.	Gender/ Age (years)		APTT (s)	FXI: C (%)	Reason for referral	Bleeding symptoms (score)	Genotype	Base variations	Amino acid variations
1	F/28		46.0	46	Check up	N	Het.	c.738G > A	Trp246*
2	F/33		50.9	53	Pre-operative screening	POB, 2	Het.	c.841 C > T	Gln281*
3	F/35		74.8	4	Antenatal examination	N	Homo.	c.738G > A	Trp246*
4	F/58		67.7	4	Check up	N	Homo.	c.738G > A	Trp246*
5	M/55		83.4	3	Check up	E, 2	Homo.	c.738G > A	Trp246*
6	M/36		76.8	2	Check up	N	Homo.	c.738G > A	Trp246*
7	F/60		98.2	3	Check up	N	Homo.	c.738G > A	Trp246*
8	F/63		84.4	3	Check up	N	Homo.	c.841 C > T	Gln281*
9	F/52		75.7	2	Pre-operative screening	N	Homo.	c.841 C > T	Gln281*
10	M/21		96.0	3	Pre-operative screening	N	Homo.	c.841 C > T	Gln281*
11	M/9		95.4	2	Check up	N	Homo.	c.841 C > T	Gln281*
12	M/43		91.8	2	Pre-operative screening	N	Homo.	c.841 C > T	Gln281*
13	F/57		91.5	4	Pre-operative screening	N	Homo.	c.1107 C > A	Tyr369*
14	F/64		121.7	2	Check up	N	Homo.	c.1107 C > A	Tyr369*
15	F/45		80.6	2	Pre-operative screening	N	Homo.	c.1325delT	Leu442Cysfs*8
16	F/58		115.4	2	Pre-operative screening	N	Homo.	c.1325delT	Leu442Cysfs*8
17	M/86		101.9	2	Pre-operative screening	N	Homo.	c.1325delT	Leu442Cysfs*8
18	F/64		59.0	13	Pre-operative screening	N	Homo.	c.1562 A > G	Tyr521Cys
19	F/44		93.9	2	Pre-operative screening	N	Comp. Het.	c.233T > C c.1253G > T	Leu78Pro^**#**^ Gly418Val
20	M/54		68.3	7	Pre-operative screening	N	Comp. Het.	c.434 A > G c.1107 C > A	His145Arg Tyr369*
21	F/55		55.1	15	Pre-operative screening	N	Comp. Het.	c.434 A > G c.1325delT	His145Arg Leu442Cysfs*8
22	F/58		107.9	2	Pre-operative screening	N	Comp. Het.	c.536 C > T c.1556G > A	Thr179Met^#^ Trp519*
23	M/36		89.2	2	Pre-operative screening	HU, 1	Comp. Het.	c.689G > T c.1556G > A	Cys230Phe^#^ Trp519*
24	F/32		73.0	10	Pre-operative screening	MH PTB, 3	Comp. Het.	c.738G > A c.938G > T	Trp246* Ser313Ile
25	F/46		89.3	6	Pre-operative screening	N	Comp. Het.	c.738G > A c.1556G > C	Trp246* Trp519Ser
26	F/55		75.6	2	Pre-operative screening	N	Comp. Het.	c.738G > A c.1556G > A	Trp246* Trp519*
27	M/63		100.6	3	Check up	PTB, 2	Comp. Het.	c.841 C > T c.1136-4delGTTG	Gln281* NA
28	F/50		86.6	2	Pre-operative screening	PTB, 2	Comp. Het.	c.841 C > T c.1136-4delGTTG	Gln281* NA
29	M/31		79.4	2	Check up	N	Comp. Het.	c.841 C > T c.1325delT	Gln281* Leu442Cysfs*8
30	F/30		49.4	17	Antenatal examination	MH, 1	Comp. Het.	c.841 C > T c.1546G > A	Gln281* Val516Met
31	F/71		54.7	3	Difficulty in hemostasis	GB, 2	Comp. Het.	c.841 C > T c.1546G > A	Gln281* Val516Met
32	F/26		101.6	4	Check up	N	Comp. Het.	c.1103G > A c.1556G > A	Gly368Glu Trp519*
33	F/19		75.6	4	Difficulty in hemostasis	MH, 2	Comp. Het.	c.1107 C > A c.1325delT	Tyr369* Leu442Cysfs*8
34	M/35		90.1	2	Check up	N	Comp. Het.	c.1107 C > A c.1325delT	Tyr369* Leu442Cysfs*8
35	M/47		84.6	2	Pre-operative screening	N	Comp. Het.	c.1107 C > A c.1556G > A	Tyr369* Trp519*
36	M/70		121.3	2	Pre-operative screening	N	Comp. Het.	c.1107 C > A c.1557G > C	Tyr369* Trp519Cys
37	F/33		66.2	2	Pre-operative screening	N	Comp. Het.	c.1107 C > A c.1562 A > G	Tyr369* Tyr521Cys
38	F/32		75.4	5	Antenatal examination	N	No genetic defect identified		
39	M/24		78.8	12	Pre-operative screening	N			
40	F/72		82.6	6	Check up	N			
41	F/57		80.1	3	Check up	N			
Reference range		29.0–43.0		82–118					

M: male; F: female; FXI: C: factor XI activity; N: no bleeding symptoms; POB: post-operative bleeding; PTB: post-traumatic bleeding; MH: menorrhagia; HU: hematuria; GB: gums bleeding; E: epistaxis; Het.: heterozygote; Homo.: homozygote; Comp.Het.: compound heterozygote

### Molecular characterization

**Fig. 1:**
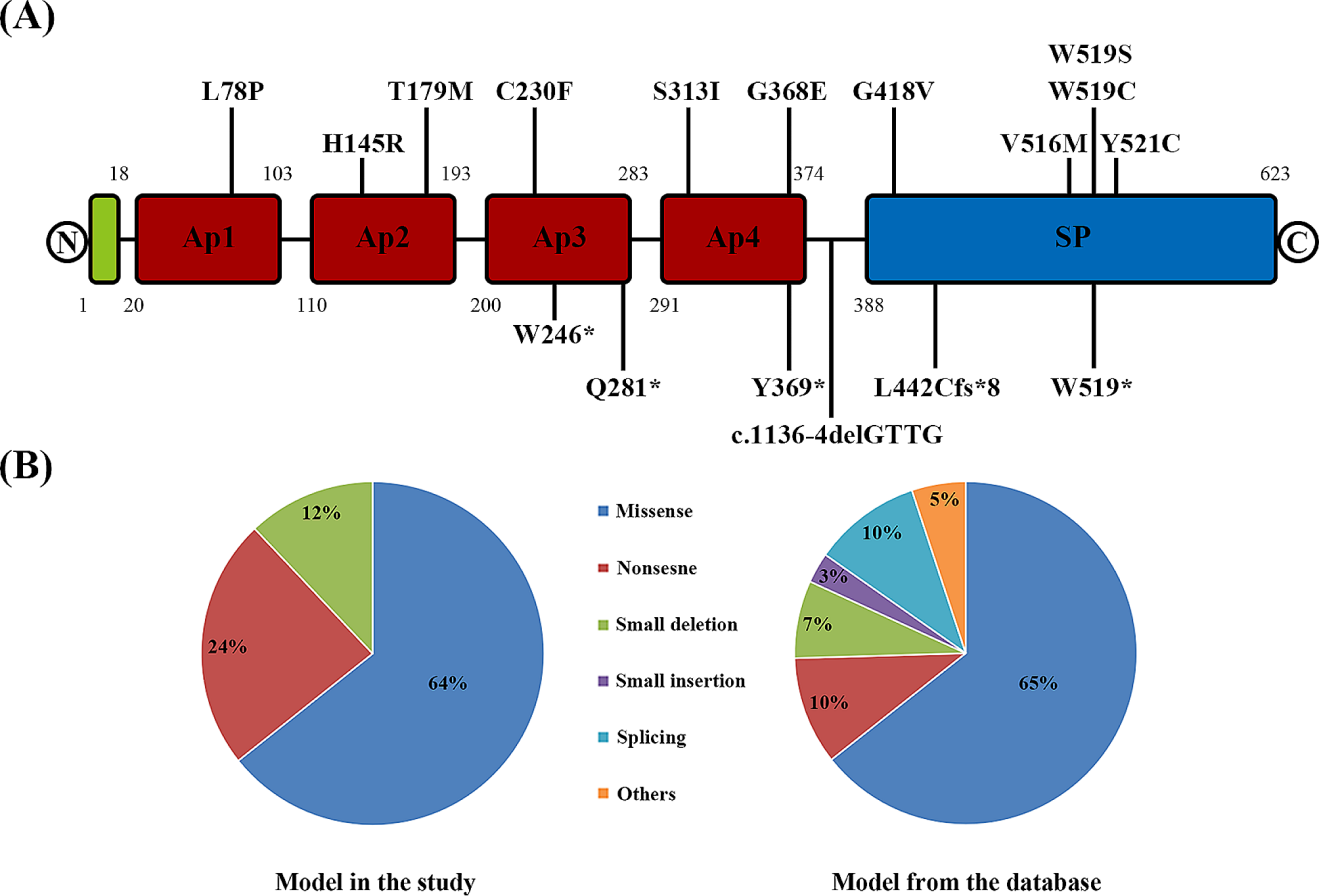
The mutational distribution and model in the cohort. (**A**) The distribution of 17 disease-causing mutations on FXI protein. The signal peptide, apple domain, and peptidase S1 are represented by the green rectangle, red rectangle, and blue rectangle, respectively. (**B**) Comparison of the mutation model in the study with the mutation model presented in the Mutation Database. Different types of mutations are indicated by different colors, respectively

Genetic analysis revealed that a total of 37 patients carried the variant, with a genetic defect rate of 90%. A total of seventeen candidate variants were identified in the *F11* gene, of which eleven were missense variants, four were nonsense variants, and two were small deletions (Table 2). As shown in Fig. 1A, variants were distributed throughout FXI, affecting the Ap1 (1), Ap2 (2), Ap3 (3), Ap4 (3), and SP (6) domains. Additionally, the deletion variant c.1136-4delGTTG occurred in intron 10. In our study cohort, the percentage of missense variants was significantly consistent compared to those documented in HGMD, but nonsense variants and small deletions were higher than in the database, and no other types of variants were identified (Fig. 1B). Of these 17 different potential variants, 6 (p.Leu78Pro, p.Thr179Met, p.Cys230Phe, p.Ser313Ile, p.Tyr521Cys, p.Leu442Cysfs*8) were reported for the first time in our laboratory.

**Table 2:** The seventeen identified variants and *in silico* analysis

Nucleotide	NP	Exon/ IVS	Type of variant	Conservation of mutant site	SIFT Score	CADD Score	POLYPHEN Score	In vitro expression	Comment/Reference
c.233T > C^**#**^	L78P	E4	Missense	Cc	Damaging 0.001	Damaging 28.9	Probably_damaging 1.0	no	CRM-
c.434 A > G	H145R	E5	Missense	Cc	Tolerable 0.444	Tolerable 9.574	Benign 0.35	yes	CRM+ [17]
c.536 C > T^**#**^	T179M	E6	Missense	Nc	Tolerable 0.115	Tolerable 0.002	Benign 0.27	yes	CRM- [17]
c.689G > T^**#**^	C230F	E7	Missense	Cc	Damaging 0.001	Damaging 27.8	Probably_damaging 1.0	no	CRM-
c.938G > T^**#**^	S313I	E9	Missense	Nc	Damaging 0.000	Damaging 22.9	Possibly_damaging 0.84	no	CRM-
c.1103G > A	G368E	E10	Missense	Cc	Damaging 0.001	Damaging 27.4	Probably_damaging 1.0	yes	CRM- [18]
c.1253G > T	G418V	E11	Missense	Cc	Damaging 0.001	Damaging 31.0	Probably_damaging 1.0	yes	CRM- [18]
c.1546G > A	V516M	E13	Missense	Cc	Damaging 0.001	Damaging 28.3	Probably_damaging 1.0	yes	CRM+ [16]
c.1556G > C	W519S	E13	Missense	Cc	Damaging 0.000	Damaging 29.8	Probably_damaging 1.0	no	unknown
c.1557G > C	W519C	E13	Missense	Cc	Damaging 0.000	Damaging 32.0	Probably_damaging 1.0	no	CRM-
c.1562 A > G^**#**^	Y521C	E13	Missense	Cc	Damaging 0.035	Damaging 29.2	Probably_damaging 1.0	no	CRM+
c.738G > A	W246*	E7	Nonsense	Not applied				no	CRM-
c.841 C > T	Q281*	E8	Nonsense					no	CRM-
c.1107 C > A	Y369*	E10	Nonsense					no	CRM-
c.1556G > A	W519*	E13	Nonsense					yes	CRM- [17]
c.1325delT^**#**^	L442Cfs*8	E12	Small del.					yes	CRM- [17]
c.1136-4delGTTG	NA	IVS10	Small del.					no	CRM-

#: novel mutations; NP: native protein; Cc: Completely conserved; Nc: Non conserved

Two, sixteen, and nineteen patients carried heterozygous, homozygous, and compound heterozygous variants, respectively (Table 1). As expected, patients who were homozygotes or compound heterozygotes presented severe FXI defects, while patients who were heterozygotes presented FXI: C values approximately half of the reference range. Of the nine patients exhibiting a hemorrhagic phenotype, one was a heterozygote, one was a homozygote, and the remaining seven were compound heterozygotes.

Most of the identified potential variants were single amino acid substitutions, nine of which occurred at completely conserved positions corresponding to those of the *F11* gene in other mammalian species (excluding p.T179M and p.S313I), demonstrating these variants may play a vital role in biological evolution. Three software programs all predicted that p.H145R and p.T179M were nonpathogenic, while all the other variants were pathogenic (Table 2). Three out of the 11 missense variants were identified as CRM+, seven were identified as CRM-, and the remaining one was uncharacterized. In addition, five variants were previously characterized through in vitro expression, including two CRM + variants (p.H145R and p.V516M) [16, 17]; two CRM- variants (p.T179M and p.G368E) exhibited a defect in the formation of FXI dimers that are not secreted outside the cell [17, 18]; and one CRM- variants (p.G418V) resulted in a reduction in plasma FXI levels via the dominant negative effect (DNE) [18]. This negative effect of the abnormal allele on the normal allele is known as the DNE.

**Fig. 2:**
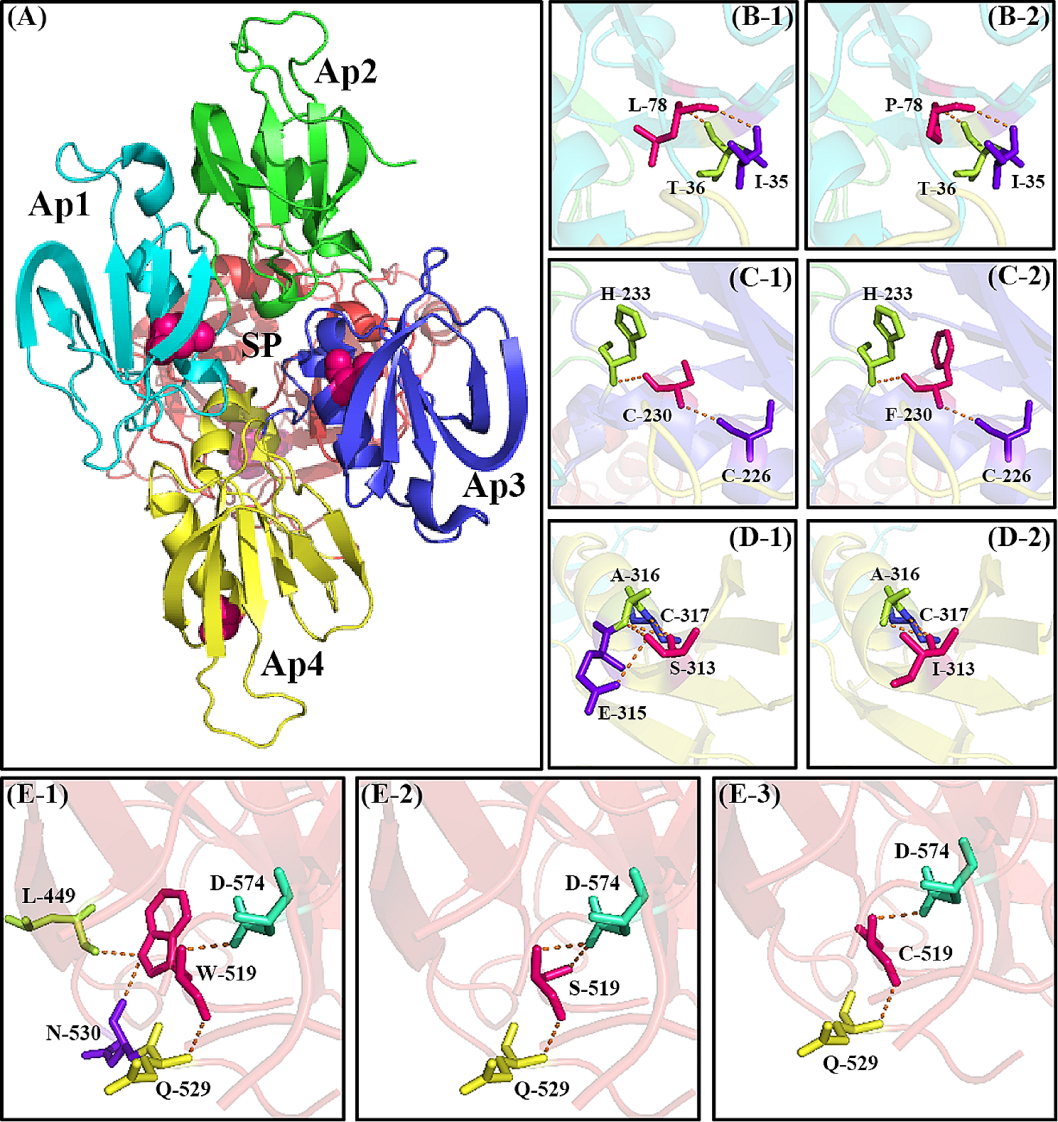
Genotype proportions, allele frequencies, and geographic origins of nine recurrent mutations. The x-axis displays the count of individuals with distinct genotypes

Protein modeling analysis was performed for the remaining five missense variants (Fig. 2). Variants substituting leucine with proline at position 78 and cysteine with phenylalanine at position 230 resulted in pronounced changes in the side chains while retaining the identical hydrogen bonds, potentially causing a disturbance in the local structural stability and intrachain disulfide bond between Cys230 and Cys236. When p.S313 was replaced with p.I313, the two hydrogen bonds that had formed with p.E315 vanished, leading to a decrease in local stability. Likewise, substituting tryptophan with a serine or a cysteine at position 519 eliminated the hydrogen bonds created by the side chain with both p.L449 and p.N530. Additionally, pedigree investigations indicated that these variants were responsible for the decreased plasma FXI.

All six null variants presented CRM- (Table 2). Two variants were studied with recombinant proteins, and they decreased plasma FXI levels by triggering nonsense-mediated mRNA decay (NMD) [17]. The c.1136-4delGTTG variant, located in intron 10, has been confirmed to be pathogenic through reverse transcription-PCR (RT-PCR) analysis due to translational retention in the intron, leading to premature stop codon generation [19].

### Recurrent variants

**Fig. 3:**
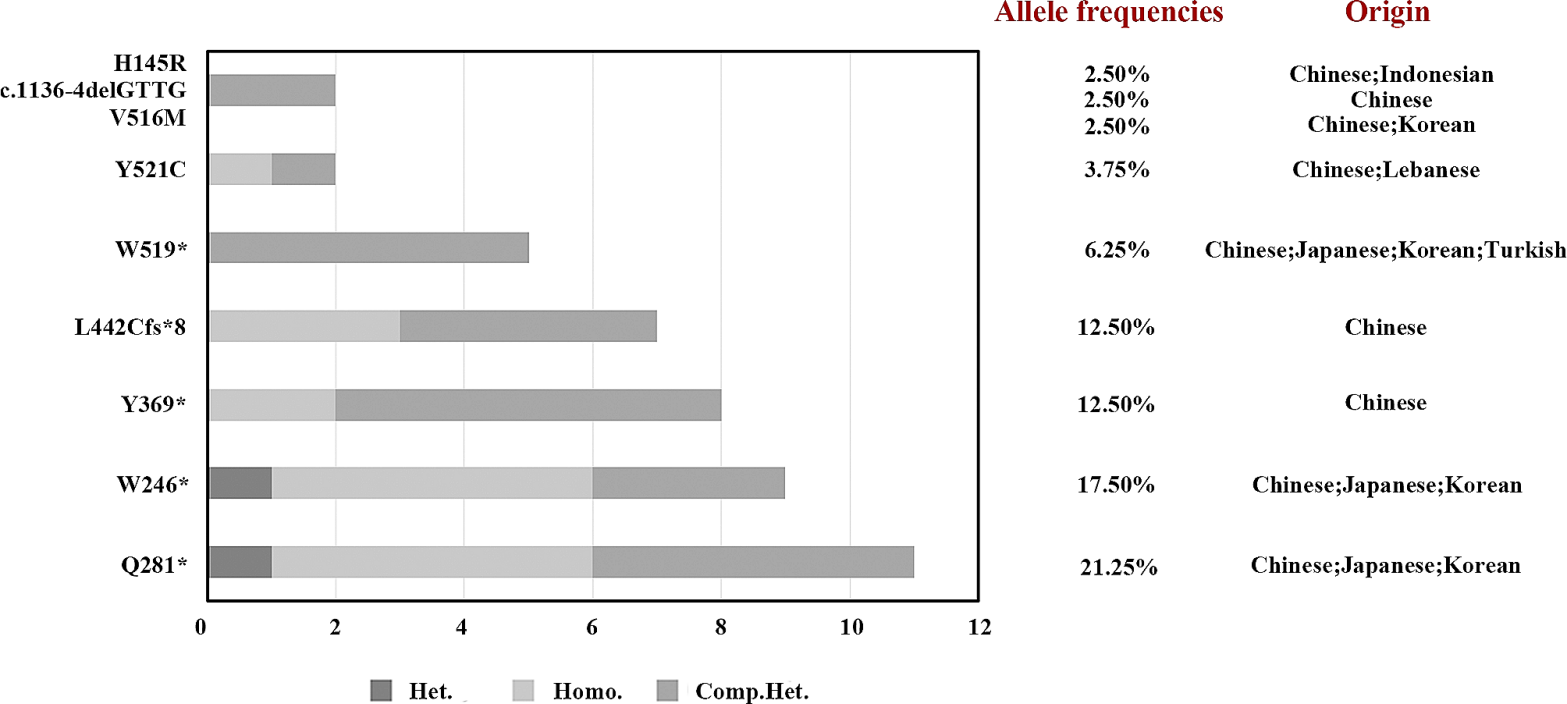
The FXI protein modeling analysis with the PyMOL (PDB: 5EOK). The five mutated amino acid residues are illustrated as hot pink spheres and sticks. The orange dashed lines represent the hydrogen bonds. (**A**) The secondary structures of the separate domains of the FXI proteins are identified through varying colors on the Cartoon, respectively. (**B**) The p.Leu78Pro variant; (**C**) The p.Cys230Phe variant; (**D**) The p.Ser313Ile variant; (**E**) The p.Trp519Ser and p.Trp519Cys variant

As shown in Fig. 3, a total of nine variants (3 missense and 6 null) appeared two or more times. The nonsense variant c.841 C > T (p.Gln281*) in 11 patients, with one heterozygote, five homozygotes, and five compound heterozygotes. Similarly, the nonsense variant c.738G > A (p.Trp246*) was detected in 9 patients, one of whom was heterozygous, five of whom were homozygous, and three of whom were compound heterozygous. These two variants had the highest allele frequencies in our cohort (21.25% and 17.50%, respectively) and have been reported in Chinese, Japanese, and Korean populations. Additionally, patients with the p.Gln281* variant exhibited a range of symptoms, from asymptomatic (*n* = 6) to mildly hemorrhagic (*n* = 5). Of the five patients with hemorrhagic symptoms, one was heterozygous, and four were compound heterozygous. Both homozygous and compound heterozygous states of the p.Trp246* variant manifested mild hemorrhagic phenotypes in one patient.

The two variants, the nonsense c.1107 C > A (p.Tyr369*) and the frameshift c.1325delT (p.Leu442Cysfs*8), had an identical allele frequency of 12.50%, which were in the homozygous or compound heterozygous states. Only one patient (P33) with p.Tyr369* and p.Leu442Cysfs*8 experienced mild bleeding. Interestingly, both of these variants have been found only in the Chinese population [20–22].

The c.1556G > A (p.Trp519*) was identified as being in a compound heterozygous state. Its allele frequency was only 6.25%, despite being reported in four different countries, specifically China, Japan, Korea, and Turkey. Only one out of five patients developed minor bleeding symptoms.

The missense variants c.1562 A > G (p.Tyr521Cys), c.1546G > A (p.Val516Met), and c.434 A > G (p.His145Arg) and the deletion variant c.1136-4delGTTG were present in only two patients. The allele frequency of the former was 3.75%, while that of the remaining three was 2.50%. Patients who carried the c.1136-4delGTTG and p.Gln281* compound heterozygous variants (P27, 28) presented abnormal post-traumatic bleeding. Furthermore, it has been determined that the c.1136-4delGTTG is exclusive to Chinese individuals. Conversely, the remaining three variants have been found in Lebanese, Korean, and Indonesian populations, in addition to Chinese populations.

## Discussion

In 1953, Robert Rosenthal [23] identified three family members with a mild hemorrhagic phenotype. Since then, there has been an increasing amount of research on congenital FXI deficiency and tentative identification of the underlying pathogenesis of the variants responsible for this deficiency. CRM- variants, typically comprising disruptive or partial missense, account for most *F11* variants, while all CRM + variants are missense [24].

In the study, only 9 out of 41 patients with FXI deficiency displayed mild bleeding, and four of nine patients experienced abnormal bleeding after hemostatic challenges. In addition, menorrhagia was the most common symptom among the women in our cohort. Thus, detecting FXI plasma levels and analyzing the *F11* gene in patients with abnormal bleeding after surgery or trauma, as well as menorrhagia, is of great value in clinical diagnosis. Potentially pathogenic variants were identified in 37 patients through Sanger sequencing. Although the four patients with severe FXI deficiency did not exhibit any candidate variants, their family history confirmed inherited FXI deficiency. Direct sequencing provides the advantages of low cost, high accuracy, and rapid detection, but it does not detect certain cryptic variants, including heterozygous large deletions or insertions, deep intronic variants in noncoding regions, or variants in promoter regions.

A total of 17 different variants were identified, with 76% and 18% of the total accounted for by CRM- and CRM + variants, respectively. Additionally, one variant could not be classified due to the absence of an FXI: Ag level and/or in vitro recombinant protein studies. FXI: Ag can be assessed through immunoturbidimetric or enzyme-linked immunosorbent assays but is not commonly carried out in most laboratories. It has been demonstrated that the CRM- variants are associated with an increased risk of pathogenicity. In contrast, CRM + variants that are composed of missense variants are less likely to be pathogenic [24]. Thus, it is imperative to distinguish between quantitative and qualitative FXI deficiency. Interestingly, three algorithms classified p.H145R and p.T179M as benign variants, despite their pathogenicity has been confirmed by in vitro expression experiments [17]. Moreover, the p.S313 was not conserved in the FXI protein across mammalian species, but it would reduce the number of hydrogen bonds that p.S313 formed with other residues, potentially resulting in unstable protein structures. Thus, caution should be taken when interpreting the results of *in silico* predictions, and comprehensive pedigree surveys and in vitro expression studies are indispensable. Notably, six variants were reported by our laboratory for the first time worldwide, providing evidence for the high allelic heterogeneity of FXI deficiency disorders. In addition, the distribution of these 17 variants across the *F11* gene further supported this conclusion. However, the prevalence of recurrent variants (9/17) led to certain exons having a high frequency of variants. For instance, incorporating exons 7, 8, and 10 into the sequencing analysis yielded a 53.57% variant detection rate (30/56). Adding exons 12 and 13 to the analysis increased the variant identification to 85.71% (48/56).

In general, congenital FXI deficiency is a rare genetic disorder with a prevalence of approximately 1 in 10^6^ individuals. However, previous genetic epidemiological studies established on the *F11* gene have shown that FXI deficiency is prevalent in East Asians, possibly as high as 29.3% of 10^6^ individuals [25]. FXI: C may not align with the clinical presentation of patients with FXI deficiency; patients with severe FXI deficiency may not exhibit abnormal bleeding, and some single heterozygous patients may still manifest a bleeding phenotype, which is in line with our cohort. Since individuals with FXI deficiency typically experience only mild to moderate hemorrhagic symptoms, routine coagulation markers such as PT and APTT are relied upon for their initial diagnosis. However, a concerning issue is that an abnormal prolongation of the APTT occurs only when the FXI: C is less than 30%. This implies that a normal APTT may exclude only severe FXI deficiency, and the remaining cases caused by heterozygous variants are often missed. It has even been reported that APTT cannot detect partial FXI defects in more than one-third of patients [26]. Upon comparing the phenotypes and genotypes of the 37 patients in the cohort, it is apparent that although the FXI: C is aligned with all the other genotypes, only two were heterozygotes. The primary reasons for referral were preoperative screening and check-ups (87.81%), which typically excluded FXI: C testing given its time and cost. Furthermore, the correlation between APTT and FXI: C in 41 FXI-deficient patients was weak, with a Spearman correlation coefficient of only − 0.581 (< 0.7). Therefore, incorporating FXI: C into routine coagulation screening is crucial for reducing morbidity and mortality in patients with FXI deficiency.

FXI deficiency is present in all ethnicities and displays a high level of allelic heterogeneity; on the other hand, certain variants exhibit noteworthy racial disparities as a result of founder effects. Reportedly, FXI deficiency was highly prevalent in the Jewish population, with up to 9% of Ashkenazi Jews presenting heterozygous variants, and more than 90% of the aberrant alleles were accounted for by the two main founder variants, specifically p.Gln135* (p.Q117*) and p.Phe301Leu (p.F283L) [27]. Additionally, p.Cys56Arg (p.C38R), p.Gln106* (p.Q88*), and p.Cys146* (p.C128*) were found to be highly prevalent in the Basque and Nantes regions of France and the British Caucasian population, respectively [28–30]. A global study of the epidemiology of *F11* gene variants revealed that the East Asian population possessed the highest heterozygote frequency; p.Q281* and p.L442Cfs*8 were variants unique to East Asians [25]. Most epidemiological studies of the *F11* gene have been conducted in Europe. It appears that, to date, there have been few epidemiological studies have focused on FXI variants in East Asia (specifically Chinese, Japanese, and Korean patients). Kim et al. [31] described 13 separate Korean patients with FXI deficiency and found that the most prevalent variants were p.Gln281* (*n* = 4) and p.Gln244* (*n* = 3), with the former showing evidence of a founder effect. Shao et al. [19] recruited 57 unrelated FXI-deficient patients from China, and they found that p.W246*, p.G416V, p.Q281*, and c.1136-4delGTTG were the four most common variants. In contrast to their study, in our cohort, p.Q281* and p.W246* were identified in 11 and 9 patients and described only in East Asians. In addition to p.Y369* and p.L442Cfs*8, which shared a 12.50% allele frequency, both of these recurrent variants have been described only in the Chinese population. In summary, we conclude that p.Q281* and p.W246* are variants specific to East Asia, while p.Y369* and p.L442Cfs*8 are specific to China that could potentially have founder effects. Unfortunately, the difficulty in preserving specimens hindered us from conducting haplotype analysis on common variants.

## Conclusion

This study presents the variant spectrum of FXI deficiency in some southeast China population and indicates a poor correlation between plasma FXI levels and clinical outcome in FXI-deficient patients. The highest allele frequency was observed in p.Q281*, p.W246*, p.Y369*, and p.L442Cfs*8. The nonsense p.Q281* was the most predominant variant, strongly suggesting a possible founder effect. Additionally, the incidence of FXI deficiency, although a rare hemorrhagic disorder, may be grossly underestimated.

## Data Availability

All data generated or analysed during this study are included in this published article. Other data used in the current study are not publicly available due to restrictions, but can be obtained from the corresponding author (wywms@126.com or wyxiehaixiao@163.com) upon reasonable request.

## References

[1] Puy C, Rigg RA, McCarty OJT (2016). The hemostatic role of factor XI. Thromb Res.

[2] Mohammed BM, Matafonov A, Ivanov I, Sun M-f, Cheng Q, Dickeson SK (2018). An update on factor XI structure and function. Thromb Res.

[3] Lewandowska MD, Connors JM, Factor XI, Deficiency (2021). Hematol Oncol Clin North Am.

[4] Hayakawa Y, Tamura S, Suzuki N, Odaira K, Tokoro M, Kawashima F (2021). Essential role of a carboxyl-terminal α-helix motif in the secretion of coagulation factor XI. J Thromb Haemost.

[5] Rimoldi V, Paraboschi EM, Menegatti M, Peyvandi F, Salomon O, Duga S (2018). Molecular investigation of 41 patients affected by coagulation factor XI deficiency. Haemophilia.

[6] Ahmadinejad M, Alavi S, Ebadi M, Rashidi A, Tabatabaei M, Rezvani A (2016). Combined high molecular weight Kininogen and factor XI deficiency. Haemophilia.

[7] Santoro C, Di Mauro R, Baldacci E, De Angelis F, Abbruzzese R, Barone F (2015). Bleeding phenotype and correlation with factor XI (FXI) activity in congenital FXI deficiency: results of a retrospective study from a single centre. Haemophilia.

[8] Wheeler AP, Gailani D (2016). Why factor XI deficiency is a clinical concern. Expert Rev Hematol.

[9] Ganor RS, Harats D, Schiby G, Gailani D, Levkovitz H, Avivi C (2016). Factor XI Deficiency protects against atherogenesis in apolipoprotein E/Factor XI double knockout mice. Arterioscler Thromb Vasc Biol.

[10] Kossmann S, Lagrange J, Jaeckel S, Jurk K, Ehlken M, Schoenfelder T, et al. Platelet-localized FXI promotes a vascular coagulation-inflammatory circuit in arterial hypertension. Sci Transl Med. 2017;9(375). 10.1126/scitranslmed.aah4923. 10.1126/scitranslmed.aah4923 28148841

[11] Sillen M, Declerck PJ. Thrombin activatable fibrinolysis inhibitor (TAFI): an updated narrative review. Int J Mol Sci. 2021;22(7). 10.3390/ijms22073670. 10.3390/ijms22073670 PMC8036986 33916027

[12] Xie H, Liu M, Zou A, Xie Y, Ye J (2021). A novel mutation (Leu60Pro) in a Chinese pedigree with hereditary factor XI deficiency. Blood Coagul Fibrinolysis.

[13] den Dunnen JT, Dalgleish R, Maglott DR, Hart RK, Greenblatt MS, McGowan-Jordan J (2016). HGVS recommendations for the description of sequence variants: 2016 update. Hum Mutat.

[14] Seligsohn U (2009). Factor XI deficiency in humans. J Thromb Haemost.

[15] Lippi G, Pasalic L, Favaloro EJ (2015). Detection of mild inherited disorders of blood coagulation: current options and personal recommendations. Expert Rev Hematol.

[16] Kuder H, Dickeson SK, Brooks MB, Kehl A, Müller E, Gailani D, et al. A common missense variant causing factor XI Deficiency and increased bleeding tendency in Maine Coon cats. Genes. 2022;13(5). 10.3390/genes13050792. 10.3390/genes13050792 PMC9140718 35627175

[17] Jiang S, Chen Y, Liu M, Zeng M, Yang L, Jin Y (2023). Gene Variants in two families with inherited coagulation factor XI Deficiency and Identification of mutations. Acta Haematol.

[18] Kravtsov DV, Wu WM, Meijers JCM, Sun MF, Blinder MA, Dang TP (2004). Dominant factor XI deficiency caused by mutations in the factor XI catalytic domain. Blood.

[19] Shao Y, Cao Y, Lu Y, Dai J, Ding Q, Wang X (2016). Clinical manifestations and mutation spectrum of 57 subjects with congenital factor XI deficiency in China. Blood Cells Mol Dis.

[20] Au WY, Cheung JW, Lam CCK, Kwong YL (2003). Two factor XI mutations in a Chinese family with factor XI deficiency. Am J Hematol.

[21] Lin F, Weng MS, Wu JR, Fang SH, Yang LY (2020). Gene variants in four pedigrees with hereditary coagulation factor XI deficiency and one novel mutation identification. Blood Coagul Fibrinolysis.

[22] Lin HY, Lin CY, Hung MH, Kuo SF, Lin JS, Shen MC (2020). Characterization of hereditary factor XI deficiency in Taiwanese patients: identification of three novel and two common mutations. Int J Hematol.

[23] Rosenthal RL, Dreskin OH, Rosenthal N (1953). New hemophilia-like disease caused by deficiency of a third plasma thromboplastin factor. Proc Soc Exp Biol Med.

[24] Dorgalaleh A, Bahraini M, Shams M, Parhizkari F, Dabbagh A, Naderi T, et al. Molecular basis of rare congenital bleeding disorders. Blood Rev. 2023;59. 10.1016/j.blre.2022.101029. 10.1016/j.blre.2022.101029 36369145

[25] Asselta R, Paraboschi EM, Rimoldi V, Menegatti M, Peyvandi F, Salomon O (2017). Exploring the global landscape of genetic variation in coagulation factor XI deficiency. Blood.

[26] Esteban J, de la Morena-Barrio ME, Salloum-Asfar S, Padilla J, Minano A, Roldan V (2017). High incidence of FXI deficiency in a Spanish Town caused by 11 different mutations and the first duplication of < i > F11: results from the Yecla study. Haemophilia.

[27] Shpilberg O, Peretz H, Zivelin A, Yatuv R, Chetrit A, Kulka T (1995). One of the two common mutations causing factor XI deficiency in Ashkenazi jews (type II) is also prevalent in Iraqi jews, who represent the ancient gene pool of jews. Blood.

[28] Zivelin A, Bauduer F, Ducout L, Peretz H, Rosenberg N, Yatuv R (2002). Factor XI deficiency in French basques is caused predominantly by an ancestral Cys38Arg mutation in the factor XI gene. Blood.

[29] Bolton-Maggs PHB, Peretz H, Butler R, Mountford R, Keeney S, Zacharski L (2004). A common ancestral mutation (C128X) occurring in 11 non-jewish families from the UK with factor XI deficiency. J Thromb Haemost.

[30] Quélin F, Trossaërt A, Sigaud M, Mazancourt PDE, Fressinaud E (2004). Molecular basis of severe factor XI deficiency in seven families from the west of France.: seven novel mutations, including an ancient Q88X mutation. J Thromb Haemost.

[31] Kim J, Song J, Lyu CJ, Kim YR, Oh SH, Choi YC (2012). Population-specific spectrum of the F11 mutations in koreans: evidence for a founder effect. Clin Genet.

